# Laparoscopic Ventral Mesh Rectopexy: Functional Outcomes after Surgery

**DOI:** 10.1055/s-0038-1675358

**Published:** 2018-10-29

**Authors:** Nasir Zaheer Ahmad, Samuel Stefan, Vidhi Adukia, Syed Abul Hassan Naqvi, Jim Khan

**Affiliations:** 1 Department of Colorectal Surgery, Queen Alexandra Hospital, Portsmouth, United Kingdom

**Keywords:** rectum, prolapse, rectopexy

## Abstract

**Aims** Rectal prolapse is a debilitating and unpleasant condition adversely affecting the quality of life. Laparoscopic ventral mesh rectopexy (LVMR) is recognized as one of the treatment options. The aim of this study was to evaluate the functional outcomes after a standardized LVMR.

**Methods** A cohort of patients who underwent LVMR from 2011 to 2015 were contacted and asked to fill questionnaires about their symptoms before and after the surgery. Three questionnaires based on measurement of Wexner fecal incontinence (WFI), obstructive defecation syndrome (ODS), and Birmingham Bowel and Urinary Symptom (BBUS) scores were used to assess the changes in postoperative functional outcomes. Some additional questions were also added to further assess bowel dysfunction.

**Results** There were 58 female patients with a mean age of 62.74 ± 15.20 (26–86) years in this cohort. About 70% of the patients participated in the study and returned the filled questionnaires. There was a significant overall improvement across all three scores (WFI: *p* = 0.001, ODS: *p* = 0.001, and BBUS: *p* = 0.001). Some individual components in the scoring systems did not improve to patient's satisfaction. No perioperative complication or conversion to an open procedure was reported in this study. Three recurrences were seen in the redo cases.

**Conclusion** LVMR is a promising way of dealing with rectal prolapse. A careful patient selection, appropriate preoperative workup, and a meticulous surgical technique undoubtedly transform the postoperative outcomes.

Rectal prolapse is a distal displacement of the rectum through the pelvic diaphragm. Graham in 1942 described it as a sliding hernia and introduced an abdominal approach for repair of the hernial defect. 1 It is analogous to the displacement of the stomach through the diaphragm resulting a hiatus hernia. 2 The stomach when displaced can be accommodated in the thorax because of the large thoracic cavity, whereas the rectum once displaced has a narrow space below the pelvic diaphragm and therefore presents as external prolapse. Like the stomach in the chest, a prolapsed rectum also produces pressure symptoms on other pelvic organs. Both conditions have a definite association with an increase in the intra-abdominal pressure. As a displaced stomach produces symptoms of reflux and in severe cases obstruction, a displaced rectum can cause fecal incontinence, obstructive defecation, or even strangulation.

The sequelae of surgical repair of both conditions are similar. The preoperative symptoms may persist after the surgery, and there may be occurrence of some new symptoms or recurrence of the condition over a variable period of time. The surgical management in both cases involves restoring the organs back to their anatomical harmony. Rectal prolapse causes a concomitant dysfunction of other pelvic organs owing to a relatively narrow pelvic cavity and an articulated anatomical arrangement. 3 4 It is believed that an optimal treatment of rectal prolapse would lead to an improvement in other organ dysfunction as well. 5 6

Different perineal and abdominal approaches have been described for surgical correction of rectal prolapse. 7 The commonly encountered complications with the perineal procedures are a reduction in rectal compliance and continence and a high recurrence rate. 8 9 10 Abdominal procedures especially with mesh placements have been reported to be associated with a definite but low rate of complications such as mesh erosion, detachment, and infection. 11

Laparoscopic ventral mesh rectopexy (LMVR) has been validated as an optimal way of dealing with this debilitating condition in a vast majority of the cases. Quality of life and functional outcome after the conventional LVMR technique described by D'Hoore et al has been replicated in the past and revealed promising results with low recurrence rates and minimal complications. 12 13 This study focused on the assessment of functional outcomes after LVMR by comparing the preoperative and postoperative values of appropriately filled, and previously validated questionnaires, by the patients.

## Methods

This was a prospective cohort study of patients presenting with a rectal prolapse and or obstructed defecation syndrome over a period of 4 years from May 2011 to September 2015.In terms of the functional disorders, patients in this cohort presented with a combination of symptoms like obstructive defecation, fecal incontinence, urgency, leakage, urinary complaints, and pelvic pain. Data on the investigations and clinical assessment were collected from the computerized radiology database and surgeon's correspondence from the outpatients. All patients had a complete history and physical examination performed at first presentation and, if indicated, a flexible sigmoidoscopy was performed on the same day. As all patients were females, a detailed obstetric history including the number of pregnancies, labor difficulties, birth weight of baby, and obstetric injuries was sought to arrange further investigations if necessary and establish a definite indication for surgery. After the clinical diagnosis of internal or external prolapse, patients had defecating proctograms, a dynamic magnetic resonance imaging (MRI) proctogram, and lower gastrointestinal endoscopy. Other investigations like anal manometry, endoanal ultrasound, and pudendal nerve studies were done selectively. The outcome measure of this study was assessment of functional outcomes before and after the surgery. Fecal incontinence was assessed using the Wexner's incontinence score, obstructive defecation was analyzed by using the obstructive defecation syndrome (ODS) score, and Birmingham Bowel and Urinary Symptoms (BBUSs) questionnaire was used to analyze bladder and bowel dysfunction. 14 15 16 Patient responses about pre- and postoperative functional outcomes were recorded and the difference between the two readings was calculated using appropriate statistical tests.

## Statistics

The preoperative and postoperative scores from the questionnaires were presented as tables. A score of zero was allocated for any unfilled question. Means and standard deviations were calculated from these values. Wilcoxon sign rank test was done to analyze the difference between preoperative and postoperative values. Ranks and ties were also analyzed for cumulative results of the three scoring systems. A *p* -value of < 0.05 was considered significant. Statistical Package for Social Sciences (SPSS) version 14 was used for calculations.

## Surgical Technique

**Fig. 1:**
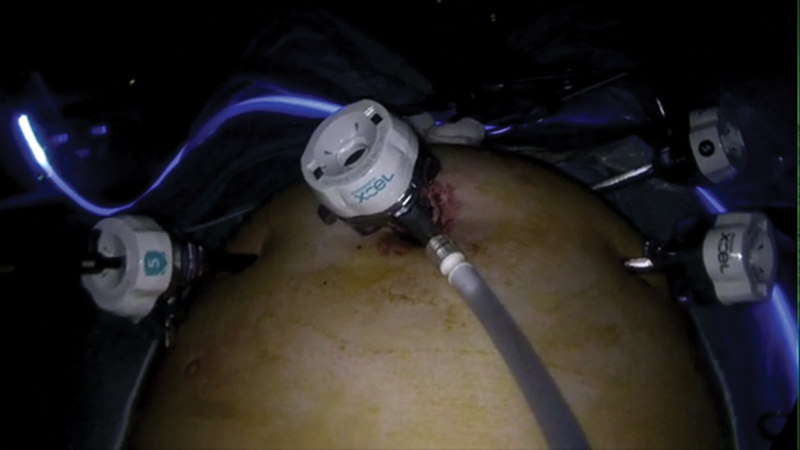
Port positioning for laparoscopic ventral mesh rectopexy.

The procedure is performed in a modified Lloyd Davis position on an antislip mattress. Four ports are used, one optical 10 port at the umbilicus, a 10- and 5-mm on the right side, and another 5-mm port in the left iliac fossa ( Fig. 1 ).

**Fig. 2:**
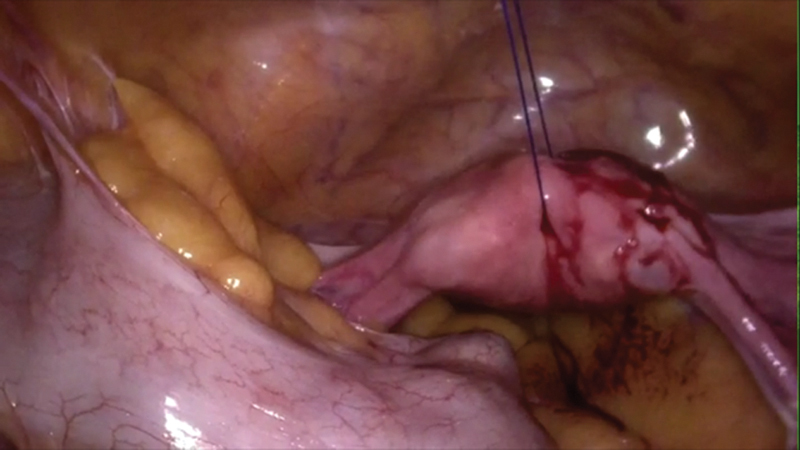
Uterus hitched for better pelvic exposure.

In the beginning of the procedure, uterus is hitched using a proline stitch on a straight needle. Sacral promontory is identified and a fold of peritoneum is stretched and dissection is started with monopolar diathermy on scissors. Once the incision is made on the stretched peritoneum, CO _2_ helps dissecting the tissue planes ( Fig. 2 ).

**Fig. 3:**
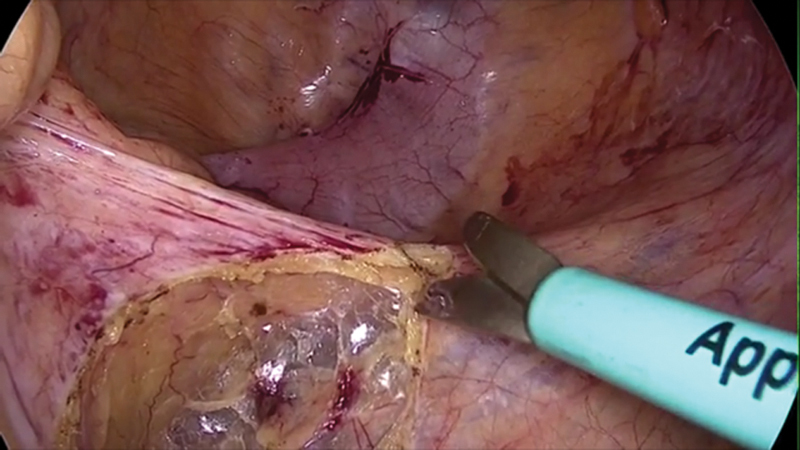
Craniocaudal dissection along the anterolateral aspect of the rectum.

A clearly defined plane preserving the hypogastric nerves can be developed in this way. Dissection is performed in craniocaudal fashion dissecting alongside the right border of the rectum ( Fig. 3 ).

**Fig. 4:**
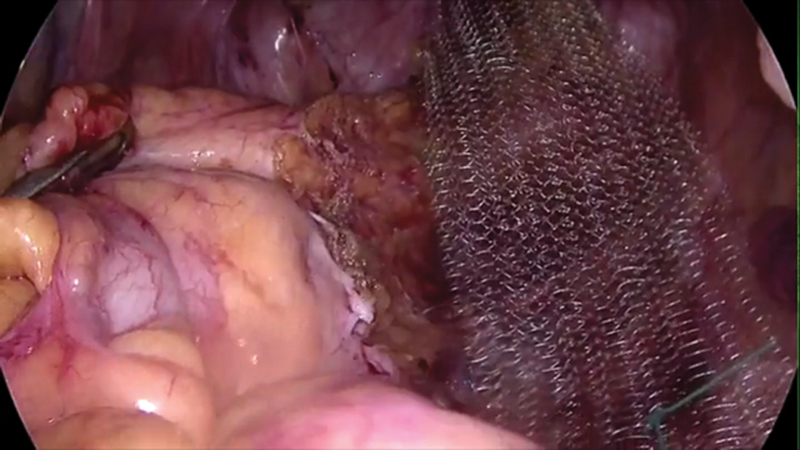
Mesh fixation.

Dissection is then extended to open the rectovaginal septum ( Fig. 4 ). The back wall of the vagina is identified and the rectum is retracted to open up the peritoneal fold.

If entered in the right plane, this plane should be avascular and there should be no damage to either the rectum or vagina. No retractors are placed in the pelvis and surgeon's left-hand instrument gives traction on the rectum and moves it to either side as needed. Dissection is continued down right to the perineal body.

**Fig. 5:**
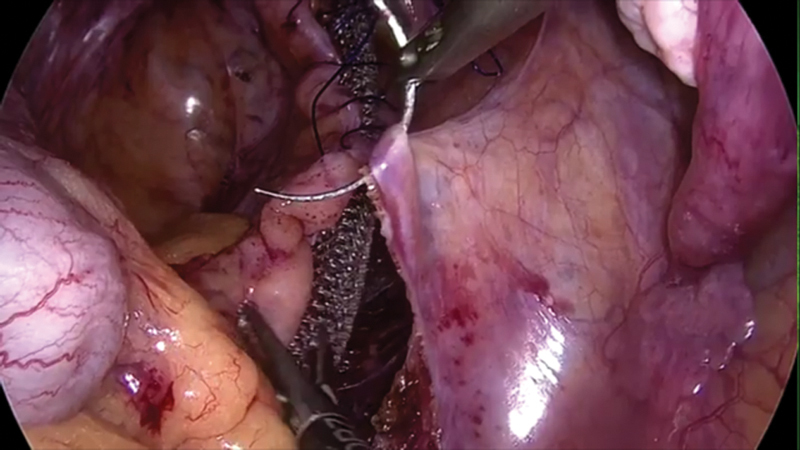
Peritoneal closure over the mesh.

At that stage, the level of dissection is assessed by holding the rectum with Johann's graspers and doing a digital rectal examination. The level of dissection should correspond to approximately 3 to 4 cm from the anal verge. An IPOM Dynamesh with a protective covering on one side and a size of approximately 5 × 15 cm is sutured to the distal end of the anterior rectal wall using four interrupted sutures of 2/0 Ethibond about 1 cm apart ( Fig. 5 ).

Care should be taken to take just enough bites and not to penetrate the rectum to prevent mesh infection. A gentle stretch is applied on the mesh to pull the rectum and fix the proximal end of the mesh to sacral promontory with tackers. Peritoneum is closed with a running suture of 2/0 polydioxanone suture covering the mesh completely.

## Results

A total of 58 patients underwent LVMR from May 2011 to September 2015. All patients were females with a mean age of 62.74 ± 15.20 (26–86) years. The mean follow-up in this series was 2.91 ± 1.18 (1–5) years. The anatomical indications for the surgery are presented in Table 1 .

**Table 1:** Indications of rectopexy

Anatomical indications for LVMR	Numbers 58
Full-thickness rectal prolapse	21
Rectorectal intussusception	15
Recurrent rectal prolapse	8
Rectocele	11
Uterus/vaginal/bladder prolapse	2

Abbreviation: LVMR, laparoscopic ventral mesh rectopexy.

The common functional disturbances that patients presented with included fecal incontinence, urinary dysfunction, ODS, dyspareunia, and pelvic pain. One of the patients passed away in the study period, therefore 57 questionnaires were sent to patients in November 2015. Thirty-nine patients responded and filled in the questionnaires giving a response rate of 70% in this study.

There was a significant improvement in the functional outcome across all three questionnaires reflecting a positive change in the quality of life after surgery. A breakdown of the scores, however, did show some derangements in some of the components in the questionnaires suggesting some dissatisfaction by the patients. The overall Wexner fecal incontinence (WFI) score after surgery improved significantly from preoperative values ( *p* = 0.001). Although there was a statistical difference between pre- and postoperative values, some patients showed dissatisfaction with the control of flatus ( *p* = 0.014). It was, however, anticipated that this group of patients would benefit from pelvic floor exercises and would be able to regain the control of flatus in due course ( Table 2 ).

**Table 2:** Wexner fecal incontinence score (0–20)

	Preoperative values		Postoperative values		Difference
Wexner	Mean	SD	Mean	SD	*p* -Value
1	1.64	1.51	0.60	1.05	0.001
2	1.51	1.52	0.59	1.04	0.001
3	2.16	1.56	1.45	1.30	0.014
4	2.08	1.96	0.92	1.56	0.002
5	2.10	1.68	0.72	1.27	0.001
Total	11.28	9.33	4.08	4.47	0.001

Abbreviation: SD, standard deviation.

Note: Q.1: How often did you lose control of a solid bowel movement?; Q.2: How often did you lose control of a liquid bowel movement?; Q.3: How often did you lose control of flatus (gas); Q.4: How often did you wear a pad because of loss of bowel control?; Q.5: How often was your life or daily routine affected by loss of bowel control?

Obstructive defecation was one of the most common symptoms in this study population. The results from the questionnaires showed a significant overall improvement postoperatively ( *p* = 0.001). Similar to the results from WFI scores analyses, some components of the ODS score did not improve to patient's expectations, and moreover, some of the components even got worse postoperatively. The use of enemata and suppositories to move the bowels got worse to some extent after the surgery ( *p* = 0.233). The probable explanation is the restoration of the anatomy with a synthetic mesh and an acute angle causing a relative decrease in propelling force to evacuate the bowels. When asked about the changes in their lifestyle, the study participants did feel a significant positive change after the surgery ( *p* = 0.003) ( Table 3) .

**Table 3:** Obstructive defecation syndrome score (0–40)

ODS	Preoperative values		Postoperative values		Difference
	Mean	SD	Mean	SD	*p* -Value
1	0.59	1.04	0.33	0.86	0.233
2	1.87	1.15	1.38	1.09	0.014
3	1.00	1.17	0.56	0.94	0.011
4	1.62	1.13	1.13	1.03	0.016
5	2.00	0.97	1.28	0.99	0.002
6	1.90	0.99	1.03	0.93	0.001
7	1.46	1.09	0.77	0.84	0.001
8	1.92	1.17	1.15	1.04	0.003
Total	13.62	8.04	7.59	5.42	0.001

Abbreviations: ODS, obstructive defecation syndrome; SD, standard deviation.

Note: Q.1: How often did you use an enema or suppository to open your bowels?; Q.2: How often did you have difficulty evacuating (i.e., passing stools that are in your back passage)?; Q.3: How often did you need to put a finger in the rectum (back passage) or the vagina to open your bowels?; Q.4: How often did you need to return to the toilet after having a bowel movement?; Q.5: How often did you feel that you have not emptied your bowels completely after having a bowel movement?; Q.6: How often did you have to strain or push to have a bowel movement?; Q.7: How much time did you need to spend on the toilet to have a complete bowel movement?; Q.8: How often did you change your lifestyle or habits because of difficulties with your bowel movements?

BBUS questionnaire-22 (BBUSQ-22) was used to assess a spectrum of quality of life indicators including bowel frequency, stool consistency, effective bowel emptying, urinary symptoms, and associated symptoms. BBUSQ-22 was split into these categories and analyzed for each component. There was a significant overall improvement in the postoperative score as compared with the preoperative values. Some categories in this questionnaire did not show a significant improvement after the surgery. These elements did raise concerns and caused some dissatisfaction among the patients ( Tables 4 , 5 , 6 , 7 , 8 ).

**Table 4:** Birmingham Bowel and Urinary Symptom score for bowel frequency

Bowel frequency	Preoperative value		Postoperative value		Difference
	Mean	SD	Mean	SD	*p* -Value
1	3.08	1.52	2.79	1.19	0.267
2	3.15	0.84	3.31	0.65	0.184
3	2.41	1.22	2.15	1.06	0.247
4	2.21	1.03	1.87	0.80	0.042

Abbreviation: SD, standard deviation.

Note: Q.1: How often did you open your bowels?; Q.2: Were your motions usual?; Q.3: Could you hold onto your motions for more than 5 minutes?; Q.4: Did you ever have to rush for the toilet to open your bowels?

**Table 5:** Birmingham Bowel and Urinary Symptom score for stool consistency

Stool consistency	Preoperative value		Postoperative value		Difference
	Mean	SD	Mean	SD	*p* -Value
1	1.92	0.80	1.54	0.75	0.005
2	1.72	0.99	1.41	0.81	0.093
3	2.44	1.14	1.74	0.78	0.001
4	2.31	1.21	1.69	0.80	0.001

Abbreviation: SD, standard deviation.

Note: Q.1: Did your stools ever leak before you could get to the toilet?; Q.2: Did you leak stools for no obvious reasons and without feeling that you wanted to go to the toilet?; Q.3: Did you have to strain to open your bowels?; Q.4: How long did you spend on the toilet, in average, for each bowel movement?

**Table 6:** Birmingham Bowel and Urinary Symptom score for bowel emptying

Bowel emptying	Preoperative value		Postoperative value		Difference
	Mean	SD	Mean	SD	*p* -value
1	2.62	1.04	2.00	1.02	0.006
2	1.95	1.14	1.51	0.88	0.005
3	1.46	0.99	1.18	0.60	0.086
4	1.97	0.95	1.64	0.74	0.038
5	2.05	1.05	1.33	0.70	0.001
6	1.38	0.54	1.79	0.61	0.003
7	2.21	1.23	2.36	1.36	0.508

Abbreviation: SD, standard deviation.

Note: Q.1: Did you feel that you could not completely empty your bowel?; Q.2: Did you use pressure or a finger to help open your bowels?; Q.3: Did you use a finger in your vagina to help you open your bowels?; Q.4: Did you have the urge to open your bowels but were unable to?; Q.5: Did you find it painful to open your bowels?; Q.6: Had you consulted a doctor in the last 6 months before your surgery about constipation?; Q.7: Did you use laxatives prior to your surgery?

**Table 7:** Birmingham Bowel and Urinary Symptom score for urinary symptoms

Urinary symptoms	Preoperative value		Postoperative value		Difference
	Mean	SD	Mean	SD	*p* -Value
1	1.74	0.99	1.59	0.85	0.244
2	2.64	1.20	2.46	1.04	0.108
3	2.05	0.85	1.95	0.88	0.415
4	1.92	1.08	1.51	0.82	0.006
5	1.97	0.81	1.67	0.83	0.027
6	1.87	0.89	1.69	0.83	0.239
7	1.49	0.82	1.18	0.50	0.005

Abbreviation: SD, standard deviation.

Note: Q.1: During the day, how many times did you urinate, on average?; Q.2: During the night, how many times did you urinate, on average?; Q.3: Did you have to rush to the toilet to urinate?; Q.4: Did you have difficulty emptying your bladder completely?; Q.5: Did urine leak before you could get to the toilet?; Q.6: Did urine leak when you were active, exerted yourself, or sneezed?; Q.7: Did urine leak for no obvious reason, and without you feeling that you wanted to go to the toilet?

**Table 8:** Associated symptoms

Associated symptoms	Preoperative value		Postoperative value		Difference
	Mean	SD	Mean	SD	*p* -Value
1	1.74	0.81	1.13	0.46	0.001
2	2.03	1.01	1.41	0.67	0.002
3	2.18	1.07	1.69	0.86	0.018
4	3.15	1.13	1.41	0.81	0.001
Total	57.10	16.72	46.90	13.30	0.001

Abbreviation: SD, standard deviation.

Note: Q.1: Did you pass any blood from your bottom?; Q.2: Did you pass any mucous from your bottom?; Q.3: Did you get pain or a dragging feeling in your pelvis?; Q.4: Did you have a lump (prolapse) coming out from your bottom?

Some additional components were incorporated into the BBSU questionnaires. These included questions about passing blood or mucous per rectum, feeling of a dragging sensation, and a prolapsing lump at the back passage ( Table 8 ). There was a significant improvement in these symptoms and the patients showed a high level of satisfaction in this regard ( Table 9 ).

**Table 9:** Ranks and ties of functional outcomes

Score system	Negative ranks	Positive ranks	Ties
Wexner	28	7	4
ODS	31	6	2
BBUSQ	30	9	0

Abbreviations: BBUSQ, Birmingham Bowel and Urinary Symptom questionnaire; ODS, obstructive defecation syndrome.

Note: Negative ranks, number of patients reporting improvement in functional outcomes; Positive ranks, number of patients reporting worsening in functional outcomes; Ties, number of patients reporting no difference in functional outcomes.

From a technical point of view, all the procedures were successfully completed laparoscopically with no conversions in this series. There was no perioperative or mesh-related complication in this cohort. However, there were three patients who had full thickness recurrence of rectal prolapse during the follow-up period. All the recurrences after LVMR were seen in patients operated for recurrence. Two of the patients were referred for biofeedback therapy. A Delorme's procedure was offered to one of the patients because of persistence of symptoms and prolapse but her symptoms resolved with conservative measures. A relatively young patient aged 46 had persistence of symptoms and was found to have mucosal prolapse 2 years after the first surgery. She required examination under anesthesia and excision of benign rectal polyps and there was no evidence of recurrence of prolapse. A 79-year-old had vaginal prolapse which was managed by the gynecologist with ring pessaries. An elderly patient had LVMR for recurrence after Delorme's procedure. She developed full-thickness recurrence after LVMR and required Altemeier's procedure with excision of the distal mesh. She had a weak pelvic floor and developed another recurrence for which she underwent Altemeier's procedure again. There was one case of intestinal obstruction secondary to incarcerated port site hernia that required surgical intervention with repair of the hernia.

## Discussion

The aim of surgical intervention is not only to restore the anatomy but also to reestablish the base line function of the organ. It is evident that restoration of the anatomy may not be achieved with perineal procedures because of inadequate exposure of the pelvic part of the rectum. However, perineal procedures still remain a suitable option for a vast majority of elderly patients with multiple comorbidities. 17 Abdominal procedures on the other hand have an advantage of sufficient exposure not only of the rectum but other pelvic organs as well.

There have been turns and twists in the evolution of abdominal procedures and a large number of surgical techniques, all claiming reasonable outcomes, have been described in the literature. 18 They all claim to cure the problem by restoring the anatomy and normalizing the physiology of the continence mechanism. But unfortunately, it has not happened with all the procedures and some approaches have even worsened the functional outcomes. 19 20 Interference with the nerves, a loss of rectal compliance, and a slow transit constipation is a probable explanation of poor functional outcome especially after resectional and posterior rectopexies. 21

A ventral mesh rectopexy has been accepted as a preferred approach because of its low recurrence rate and better functional outcomes. 22 Unlike the antireflux procedures for gastroesophageal reflux disease which are tailored according to the anatomical abnormality encountered at the time of surgery and range from different degrees of fundoplications and repair of the crural defect with or without a mesh, a ventral mesh rectopexy is more or less an identical procedure for almost everyone and involves fixing a mesh anteriorly to the lower rectum and posteriorly to the sacral promontory without posterior mobilization of the rectum. A laparoscopic approach for ventral mesh rectopexy has been recognized as the standard of care to deal with the rectal prolapse. 23

In the current literature, LVMR is considered a gold standard treatment for elective repair of rectal prolapse. 24 25 26 A couple of Cochrane reviews have further validated these findings. 27 28 A step further has been taken by exhibiting the procedure successfully in emergency situations. 29 Because of the minimal morbidity and quick recovery after the surgery, enthusiasts are even recommending it as a day case procedure. 30 The safety and the popularity of the laparoscopic procedure has attracted the attention of robotic surgeons with several robot-assisted rectopexies performed successfully. 31 These are the areas in evolution and would need further endorsements before being implemented in regular practice.

As with any other surgical procedure, LVMR has potential complications as well. These include functional and mesh-related complications. The mesh-related complications include erosion and intrarectal mesh migration leading to fistula formation. 32 33 Development of high-grade hemorrhoids has also been recognized as a complication of LVMR and it may act as a precursor of recurrence of rectal prolapse. 34 No mesh-related complications were seen after a follow-up of 4 years in this series. It is emphasized that it is the right surgical technique which prevents things going wrong in most of the cases.

As mentioned earlier, with the restoration of rectal anatomy, functional outcomes such as ODS, incontinence, and bladder dysfunction improves as well. The functional outcomes in this study were assessed by means of questionnaires asking patients to rate the WFI score, ODS score, and BBUS score before and after the surgery. Although the response rate was relatively low, yet the results showed a significant improvement in the quality of life parameters in most of the cases.

The functional outcomes keep getting better or worse in the postoperative period. A single measurement of the function after surgery may not be a true reflection of the postoperative change and is therefore one of the limitations of this study. A relatively lower response rate to fill the questionnaires by the patients constitutes another limitation of the study. A lack of regular postoperative clinical assessments and the absence of a questionnaire about the sexual function are other limitations which could have changed the overall findings of this study.

This study analyzed the postoperative subjective outcomes assuming that no disturbance in the postoperative anatomy has occurred unless it was very obvious clinically or had been reported by the patient. Minor rectoceles and sometimes internal intussusceptions may be completely subclinical and asymptomatic and may get unnoticed in the postoperative period. It is proposed that future studies may use dynamic MRI as an objective measure to diagnose early recurrence in the follow-up period.

## Conclusion

LVMR is favored for repair of rectal prolapse and pelvic floor dysfunction because of low recurrence rate and low incidence of postoperative complications. The recurrence rate in this series was the same as reported in the literature and there was no mesh-related complication. Most of the recurrences in this study were seen in the cases reoperated for recurrent prolapse. A careful selection of patients, vigorous preoperative workup, and a meticulous surgical technique are recommended for the management of this debilitating condition.
